# Risk of Myocarditis and Pericarditis among Young Adults following mRNA COVID-19 Vaccinations

**DOI:** 10.3390/vaccines10050722

**Published:** 2022-05-05

**Authors:** Abdallah Alami, Daniel Krewski, Donald Mattison, Kumanan Wilson, Christopher A. Gravel, Paul J. Villeneuve, Patrick J. Farrell, James A. G. Crispo, Santiago Perez-Lloret

**Affiliations:** 1 School of Mathematics and Statistics, Carleton University, Ottawa, ON K1S 5B6, Canada; pfarrell@math.carleton.ca; 2 School of Epidemiology and Public Health, University of Ottawa, Ottawa, ON K1S 5B6, Canada; dkrewski@uottawa.ca (D.K.); dmattison@risksciences.com (D.M.); cgravel@uottawa.ca (C.A.G.); 3 Risk Sciences International, Ottawa, ON K1P 5J6, Canada; 4 Arnold School of Public Health, University of South Carolina, Columbia, SC 29208, USA; 5 Department of Medicine, University of Ottawa, Ottawa, ON K1R 6M1, Canada; kwilson@toh.ca; 6 Bruyère Research Institute, Ottawa, ON K1R 6M1, Canada; 7 Ottawa Hospital Research Institute, Ottawa, ON K1R 6M1, Canada; 8 Department of Epidemiology, Biostatistics and Occupational Health, McGill University, Montreal, QC H3A 0G4, Canada; 9 School of Mathematics and Statistics and Department of Neurosciences, Faculty of Science Carleton University, Ottawa, ON K1S 5B6, Canada; paul.villeneuve@carleton.ca; 10 Faculty of Pharmaceutical Sciences, University of British Columbia, Vancouver, BC V6T 1Z4, Canada; jcris021@uottawa.ca; 11 Division of Human Sciences, Northern Ontario School of Medicine, Sudbury, ON P3E 2C6, Canada; 12 Data Science Research Laboratory, Pontificia Universidad Católica Argentina, Buenos Aires C1107AFD, Argentina; splloret@gmail.com; 13 Departamento de Fisiología, Facultad de Medicina, Universidad de Buenos Aires, Buenos Aires C1121ABG, Argentina

**Keywords:** vaccine adverse event reporting system (VAERS), COVID-19, myocarditis, pericarditis, passive surveillance, vaccine safety

## Abstract

There have been reports of cases of myocarditis and pericarditis as rare complications following mRNA COVID-19 vaccinations among young adults. While most reported cases are mild, this potential vaccine safety signal should be closely monitored. Using data from the CDC and the Vaccine Adverse Event Reporting System (VAERS), we calculated the combined reporting rate of myocarditis and pericarditis stratified by age group, sex, vaccine dose, and manufacturer, and compared these rates to the crude background incidence rates. Compared to the general population prior to the administration of the first COVID-19 vaccines in December 2020, we identified a higher-than-expected reporting rate of myocarditis and pericarditis following mRNA vaccination; the risk was higher after a second vaccine dose, higher in males than in females, and decreased with age. The highest risk was seen in males 12–17 years of age with approximately 6 cases per 100,000 second doses. Our findings suggest an increased risk of myocarditis and pericarditis in young males following a second dose of an mRNA COVID-19 vaccine. Since these findings are based on safety signals derived from passive surveillance data, confirmatory epidemiological studies should be undertaken.

## 1. Introduction

Recently, there have been increasing reports of cases of young males with inflammation of the heart muscle—myocarditis—and/or inflammation of the outer sac which contains the heart—pericarditis—following mRNA COVID-19 vaccinations with Pfizer-BioNTech (Comirnaty) or Moderna (Spikevax) [1,2,3,4]. Symptoms usually begin within days of vaccination, and range from dyspnea, chest pain, fatigue, heart palpitations, to syncope. Suspected cases of cardiac inflammation are followed by a cardiologist for further evaluation and, while the severity and symptoms vary from mild to severe, most cases that receive medical care recover completely within days [1]. Historically, adverse cardiac events such as myocarditis and pericarditis have rarely been reported following vaccination: documented cases were mainly among young males following vaccination against smallpox, influenza, and/or hepatitis B [5].

In the United States (US), the Centers for Disease Control and Prevention (CDC) has closely monitored these and other adverse events following immunization (AEFIs). Although the total number of adverse events for mRNA COVID-19 vaccines is small compared to nearly 500 million vaccine doses administered to date [6], the agency has recently issued guidance on the occurrence of myocarditis following vaccination, stating that the benefits of vaccination outweigh the risks of rare cardiac complications. While still recommending vaccination for adolescents as young as 12 years of age, the CDC advises individuals who experience myocarditis and/or pericarditis after their first mRNA COVID-19 vaccine to defer getting their second dose until additional data are available [1]. However, a recent study noted that most individuals under 21 years of age who develop myocarditis after an mRNA COVID-19 vaccination have minor clinical manifestations with quick remission of symptoms [7]. Using data from the CDC and the Vaccine Adverse Event Reporting System (VAERS), we evaluated the potential risks of myocarditis and/or pericarditis following mRNA COVID-19 vaccines among males and females 12 years of age and older.

## 2. Materials and Methods

All reports of adverse reactions to mRNA COVID-19 vaccines between 14 December 2020 and 9 July 2021 were abstracted from VAERS. For each report, signs and symptoms of adverse events were coded using the Medical Dictionary for Regulatory Activities (MedDRA), which provides clinically validated standardized terminology for such events [8,9].

Extracted data were analyzed at the preferred term level using MedDRA version 23.0 for AEFI reports containing any of the prespecified terms “myocarditis”, “pericarditis”, or “myopericarditis” [9]. While MedDRA preferred terms may not always represent a medically confirmed diagnosis, and a reported AEFI can be assigned multiple MedDRA terms, all identified cardiac-related AEFI reports were examined for symptoms that met the CDC’s case definition of probable myo/pericarditis: a probable case requires “the presence of at least one of the clinical symptoms (acute chest pain, pressure, discomfort, palpitations, syncope, dyspnea, shortness of breath, or pain with breathing) and at least one abnormal testing (elevated troponin, ECG or EKG, abnormal cardiac function, or abnormal findings on Magnetic Resonance Imaging), without any other identifiable cause.” [10] These criteria are specifically used for surveillance purposes only, and pertain to patients who have clinically suspected symptoms of myocarditis, pericarditis, or both [10]. Prior to analysis, data cleaning and deduplication were carried out. As 92% of patients experience onset of symptoms within 7 days of vaccination [11], only events that occurred in the US and with time to onset of symptoms within a 7-day window following vaccination were included in our analyses; we excluded reports with missing vaccine manufacturer (0.22%), dose number (20.3%), or patient age (3.04%).

Using the CDC’s COVID Data Tracker for trends in COVID-19 vaccinations and national US COVID-19 vaccination demographics, we were able to estimate the observed reporting rate of myo/pericarditis following mRNA vaccination based on the total number of AEFI reports received by VAERS as the numerator, divided by the total number of COVID-19 doses administered during the same period as the denominator [12]:

$$Observed\;reporting\;rate=\frac{Total\;number\;of\;AEFI\;reports\;received\;by\;VAERS\;}{Total\;number\;of\;COVID-19\;vaccine\;doses\;administered\;}$$

Reporting data were queried for each age group to determine the total number of doses given, the number of individuals that received at least one dose, and the number of individuals who completed the two-dose series. The proportion of doses administered for males and females within each age group were inferred from data published by the CDC’s Advisory Committee on Immunization Practices as of 11 June 2021, assuming these proportions remained constant through 9 July 2021, and were the same for both the Pfizer-BioNTech and Moderna vaccines [13]. Reporting rate estimates, expressed as cases per million vaccines administered, were calculated and stratified by age group, vaccine dose (first/second), and vaccine manufacturer, and then compared to the background rate of occurrence of myo/pericarditis.

Although the annual incidence of myocarditis in the US has been widely reported as being between 1 and 10 per 100,000 [14,15,16,17], the true incidence of myocarditis and pericarditis remains somewhat uncertain [18]. Although Willame et al., 2021 [19] estimated the incidence of myo/pericarditis in the US to be 3.13 per 100,000 person years using data from the US Vaccine Safety Datalink (VSD) reported by Kuntz et al., 2018 [20], this result is based on only 10 cases. A recent study based on electronic health records and administrative claims in five European countries (Denmark, Italy, Netherlands, Spain, UK) supported by the European Medicines Agency (EMA) suggests an incidence rate of 1 to 20 cases per 100,000 person-years ([19], Figure 30), which scales to 0.19 to 3.84 cases per million persons within a 7-day period. With a male:female case ratio of 1.6:1.0 [21], the expected background rate of myo/pericarditis ranges from 0.12 to 2.36 per million for males and from 0.07 to 1.47 per million for females. These results provide a crude range for the background myo/pericarditis in the general US population prior to COVID-19 vaccination, which can serve as a benchmark for evaluating the observed rates of myo/pericarditis following vaccination reported in the present study.

## 3. Results

During the analysis period, data from the CDC indicated that nearly 318 million mRNA COVID-19 vaccine doses were administered in the US, of which 184 million were Pfizer-BioNTech and 134 million were Moderna. Throughout this period, 15 million doses of Pfizer-BioNTech were administered to individuals between 12–17 years of age, while individuals 18–29 years of age received approximately 25 million Pfizer-BioNTech and 20 million Moderna doses, respectively. During the same period, the VAERS database received a total of 1106 AEFI reports on suspected cases of myocarditis and 674 reports on pericarditis following mRNA vaccination [22].

Of the 1780 AEFI reports on myo/pericarditis, 60% were observed among individuals less than 29 years of age, while 75% were classified as serious, which as defined in VAERS, are reports with at least one of the following reported outcomes: healthcare provider clinic visit (5.3%), emergency room visit (12.8%), life threatening (10.2%), hospitalization (66%), extended hospitalization if the vaccine was administered while the patient was already hospitalized (0.9%), and permanent disability (4%) [14]. Although death was mentioned in 0.8% of the AEFI reports, it is not clear that vaccination was the underlying cause. Among cases for which the vaccine dose sequence (first or second) was reported, 32% of reported myo/pericarditis cases occurred following the first dose, and 68% occurred after the second dose. Myo/pericarditis was more commonly reported among males than females: 60% of the cases following the first dose and 80% of cases following the second dose were among males.

Table 1 summarizes the observed and expected reporting rates of myo/pericarditis stratified by vaccine type, vaccine dose, and by vaccine recipient sex, expressed per million doses administered. Reporting rates for myo/pericarditis estimated for each age group revealed the highest risks in the 12–17 and 18–29-year-old age groups. Among males 12–17 years of age, the observed number of cases was higher than expected following a first Pfizer-BioNTech dose (15 reports of myo/pericarditis per million doses compared with a maximum of 2.4 cases expected), and markedly higher than expected following the second vaccine dose (66 reports per million doses, corresponding to a risk between 6.4 and 6.6 per 100,000 doses, considering the range in the expected background rate of occurrence of myo/pericarditis) [19,20]. For females within the same age group, the observed number of cases was slightly above the upper limit of the expected range following a first Pfizer-BioNTech dose, but was notably higher than expected among individuals who received the second mRNA vaccine dose. Although the Moderna vaccine is not yet approved by the US Food and Drug Administration (FDA) for individuals younger than 18 years of age, a higher-than-expected number of cases was observed among males 18–29 years of age after both a first and second dose, with a risk of approximately 3 cases per 100,000 doses after the second dose in this age group). The risk of myo/pericarditis associated with both the Pfizer-BioNTech and Moderna vaccines decreased with age in both males and females after both the first and second doses.

## 4. Discussion

To establish a national early warning system for potential vaccine safety signals, the CDC and FDA co-established VAERS in 1990 [22]. This passive surveillance system for US licensed vaccines accepts spontaneous and voluntary reports of vaccine adverse events from different stakeholders such as vaccine manufacturers, vaccine recipients, military, and healthcare providers, who are mandated to report any adverse events that are listed in the VAERS Table of Reportable Events Following Vaccination if the event occurred within the specified time period [22,23].

After stratifying by sex and age group, the present vaccine-safety surveillance analysis of VAERS data identified a possible association between mRNA COVID-19 vaccines and the incidence of myo/pericarditis among young males aged 12–29 years of age. Myo/pericarditis reporting rates were similar between both mRNA COVID-19 vaccines (Moderna and Pfizer-BioNTech) across the different age groups and are consistent with other studies. Similar results have recently been reported by Oster et al. (2022), who also used myocarditis reports in VAERS following mRNA COVID19 vaccination, in comparison with background rates using 2017–2019 claims data from the IBM MarketScan Commercial Research Database [24]. Other studies including different study designs and involving electronic and administrative health records have also found evidence of an increased risk of myocarditis and pericarditis among younger males receiving two doses of mRNA vaccine [25,26,27,28], consistent with the present findings. Overall, the observed reporting rate of myo/pericarditis for males and females across all ages combined, including both first and second doses of the two mRNA vaccines, was 5.59 cases per million doses (1780 reported cases/318 million total mRNA vaccine doses), essentially equal to the rate of 5.98 per million doses reported in an earlier study using VAERS data [29].

Although the etiology of this mRNA vaccine-associated adverse event is still not well-understood, with many asymptomatic cases unrecognized or not reported, verifying possible vaccine-linked cases is clinically important. Currently, there is no confirmed causal link between COVID-19 mRNA vaccination and myo/pericarditis, although different contributing factors and plausible biological mechanisms have been hypothesized.

One of these postulated theories suggests a dysregulated hyperactivated immune response following mRNA vaccination [30,31,32]. As children and adolescents usually have a more robust immune response than the elderly, the possible involvement of autoimmunity in inflammatory myo/pericarditis might help us understand why these population groups demonstrate a greater rate of reporting post-vaccination myo/pericarditis, possibly in genetically predisposed individuals [33,34]. Another theory is based upon the hypothesis of the immunogenicity of mRNA. Many types of mRNA vaccines have been developed during the last two decades, with some showing tremendous promise to combat cancer and other viral illnesses [35]. Nonetheless, the ability to overcome the immunogenicity of mRNA, which can induce proinflammatory cytokines, was a difficult challenge. The development of different carrier-based mRNA vaccines represents an important advance, as they may reduce the excessive innate immunogenicity, instability, and inefficiency of the vaccine’s mRNA components [34,36]. However, it has been postulated that molecular mimicry and different components of mRNA vaccines might still be the underlying cause of post-vaccination inflammation and cardiac response [37].

Although the underlying etiology of male dominance in reporting post-vaccination myo/pericarditis remains unclear, proposed pathophysiological mechanisms include the role of sex hormones and their effect on the immune and cardiovascular systems, and their likely involvement in inflammatory cardiomyopathy [38,39]. The expression of pro- and anti-inflammatory signaling pathways can be highly regulated by sex hormones, which can impact the severity of cardiac-muscle inflammation [39]. Nevertheless, the significance of sex hormones on vaccine-related acute myocarditis is still not well understood. While estrogen has a complex immunomodulatory role in females, it exhibits an anti-inflammatory protective effect by lowering the levels of autoantigen-specific proinflammatory molecules [39,40,41,42]. On the other hand, testosterone plays an opposite role in men, as it directly promotes proinflammatory receptors, inhibiting anti-inflammatory cells, and upregulating cardiac fibrotic remodeling genes [43,44,45].

Although reporting rates of myo/pericarditis are elevated in adolescents and young adults, evidence from a recent cohort study where investigators simultaneously evaluated the risk of vaccine adverse events among vaccinated individuals and the risk of the same adverse event among individuals who contracted COVID-19 infection revealed that COVID-19 infection is itself a higher risk factor for myocarditis in this population, and significantly increases the risk of multiple other serious adverse events [46,47].

While VAERS offers real-world evidence on vaccine safety, it is subject to certain limitations. This system is known to be susceptible to reporting bias, such as stimulated reporting induced by public awareness and media attention or, conversely, incomplete reporting in the absence of public awareness of a possible vaccine safety concern. Spontaneous AFEI reports may also be subject to variable quality, inconsistency, and substantial proportions of missing data [48,49,50,51]. Another drawback is the inability to exclude reports of previously COVID-19 infected patients who developed myo/pericarditis after getting the vaccine or to compare post-vaccination adverse event rates to unvaccinated persons or individuals treated for COVID-19 illness. Despite these limitations, our study adds to the limited data on the risk of myo/pericarditis following mRNA vaccination.

## 5. Conclusions

The present analysis of VAERS reports of myo/pericarditis following one or two doses of mRNA COVID-19 vaccine revealed an increase in the number of reported myo/pericarditis following mRNA second dose injections among adolescents and younger males. Although the time to onset, age, and sex differences in AEFI prevalence coincide with the clinical manifestations of myo/pericarditis, additional epidemiological studies are needed to further assess the nature of this rare vaccine safety signal and to enhance diagnosis and active monitoring. Considering the potentially serious complications of COVID-19 infections in children and young adults, ranging from long-term effects to multi-system syndrome, the risk-benefit assessment for vaccination remains favorable, with the benefits of vaccination outweighing any risk of vaccine-associated myo/pericarditis.

## Figures and Tables

**Table 1:** Number of cases of myo/pericarditis per million mRNA vaccine doses, compared to expected rates in the US general population stratified by age, sex, vaccine dose (first or second), and vaccine type.

Sex	Number of Doses/ Observed No. of Cases	Age (Years)						
		12–17	18–29	30–39	40–49	50–64	65+	All
**Pfizer-BioNTech (** **Comirnaty)**								
**Males**	Number of first doses	4,130,345	6,416,041	6,274,534	6,307,233	11,537,946	11,593,304	46,259,406
	Observed no. of cases	62	51	25	10	16	18	182
	Observed no. of cases per million doses *	**15.0**	**7.9**	**4.0**	1.6	1.4	1.6	3.9
	Number of second doses	3,105,286	5,158,756	5,212,165	5,354,938	9,999,709	10,237,134	39,067,988
	Observed no. of cases	205	180	53	37	25	17	517
	Observed no. of cases per million doses *	**66.0**	**34.9**	**10.2**	**6.9**	**2.5**	1.7	13.2
**Females**	Number of first doses	4,370,879	7,474,687	6,962,009	7,197,081	13,146,233	14,065,052	53,215,944
	Observed no. of cases	12	15	16	13	25	15	96
	Observed no. of cases per million doses *	**2.7**	**2.0**	**2.3**	**1.8**	**1.9**	1.1	1.8
	Number of second doses	3,333,409	6,229,183	5,968,924	6,197,721	11,500,977	12,318,105	45,548,323
	Observed number of cases	28	27	16	25	28	10	134
	Observed no. of cases per million doses *	**8.4**	**4.3**	**2.7**	**4.0**	**2.4**	0.8	2.9
**Moderna (Spikevax)**								
**Males**	Number of first doses	NA	5,362,926	4,858,349	5,086,879	9,161,298	9,530,389	33,999,843
	Observed no. of cases		47	20	13	17	11	108
	Observed no. of cases per million doses *		**8.8**	**4.1**	**2.6**	1.9	1.2	3.2
	Number of second doses	NA	4,364,363	4,024,907	4,279,450	7,919,481	8,335,937	28,924,140
	Observed no. of cases		137	44	20	18	20	239
	Observed no. of cases per million doses *		**31.4**	**10.9**	**4.7**	2.3	2.4	8.3
**Females**	Number of first doses	NA	5,675,241	5,659,976	5,644,228	10,453,808	10,858,841	38,292,095
	Observed no. of cases		15	16	6	17	14	68
	Observed no. of cases per million doses *		**2.6**	**2.8**	1.1	1.6	1.3	1.8
	Number of second doses	NA	4,684,982	4,860,065	4,900,788	9,165,884	9,587,422	33,199,143
	Observed number of cases		16	8	16	22	11	73
	Observed no. of cases per million doses *		**3.4**	**1.6**	**3.3**	**2.4**	1.1	2.2

* Observed no. of cases per million doses outside of expected range 0.12–2.36 for males and 0.07–1.47 for females in the general population prior to the administration of the first COVID-19 vaccines in December 2020 are marked in **bold** type. Background rates of myo/pericarditis are based on results observed in multiple studies synthesized by Willame et al. [19]. Abbreviations: N/A, not applicable (Moderna COVID-19 vaccine not approved for this age group as of 31 July 2021).

## Data Availability

All data on reports of myo/pericarditis submitted to the U.S Food and Drug Administration’s Vaccine Adverse Events Reporting System are available for download from: https://vaers.hhs.gov/data/datasets.html (accessed on 20 July 2021). Data on the number of first and second doses of mRNA COVID-19 vaccines during the study period are given in the U.S. Centers for Disease Control and Prevention’s Data Tracker, available at https://www.cdc.gov/coronavirus/2019-ncov/vaccines/distributing/demographics-vaccination-data.html (accessed on 24 July 2021). Data on mRNA doses by age and gender are given in reference [14], available at: https://www.cdc.gov/vaccines/acip/meetings/downloads/slides-2021-06/03-COVID-Shimabukuro-508.pdf.
